# Supplementary material for: Gender and Sex in Medical Practice: An Exploratory Study on Knowledge, Behavior, and Attitude among Sicilian Physicians
Source: Int J Environ Res Public Health. 2023 Jan 1;20(1):827. doi: 10.3390/ijerph20010827 (PMC9819832; doi:10.3390/ijerph20010827)
Supplement: Supplementary file 1 [file ijerph-20-00827-s001.zip › ijerph-2049097-supplementary.pdf]

Supplementary Tables

Table S1. *Participants' answers on ITEM#1, by medical specialty group and sex.*

		Female		*Total female*	*Male*		*Total male*	*TOTAL*
		NO	*YES*		*NO*	*YES*		
Group I	n	3	4	7	1	3	4	11
	%	42,9	57,1	63,6	25,0	75,0	36,4	
Group II	n	4	21	25	0	6	6	31
	%	16,0	84,0	80,6	0,0	100,0	19,4	
Group III	n	1	16	17	7	23	30	47
	%	5,9	94,1	36,2	23,3	76,7	63,8	
Group IV	n	9	6	15	5	18	23	38
	%	60,0	40,0	39,5	21,7	78,3	60,5	
Group V	n	6	3	9	8	9	17	26
	%	66,7	33,3	34,6	47,1	52,9	65,4	
Group VI	n	44	90	134	32	92	124	258
	%	32,8	97,2	51,9	25,8	74,2	48,1	
Group VII	n	14	40	54	9	22	31	85
	%	25,9	74,1	63,5	29,0	71,0	36,5	
TOTAL	**n**	**81**	**180**	**261**	**62**	**173**	**235**	**496**
	**%**	**31,0**	**96,0**	**52,6**	**26,4**	**73,6**	**47,4**	

Table S2. *Participants' answers on ITEM#2, by medical specialty group and sex.*

		Female			*Total female*	*Male*			*Total male*	*TOTAL*
		NO	*MAYBE*	*YES*		*NO*	*MAYBE*	*YES*		
Group I	n	0	0	7	7	0	1	3	4	11
	%	0,0	0,0	100,0	63,6	0,0	0,0	100,0	19,4	
Group II	n	0	2	23	25	0	0	6	6	31
	%	0,0	8,0	92,0	80,6	0,0	0,0	100,0	19,4	
Group III	n	0	1	16	17	2	2	26	30	47
	%	0,0	5,9	94,1	36,2	6,7	6,7	86,7	63,8	
Group IV	n	0	5	10	15	0	3	20	23	38
	%	0,0	33,3	66,7	39,5	0,0	13,1	87,0	60,5	
Group V	n	2	1	6	9	0	1	16	17	26
	%	22,2	11,1	66,7	34,6	0,0	5,9	94,1	65,4	
Group VI	n	5	12	117	134	5	8	111	124	258
	%	3,7	9,0	87,3	51,9	4,0	6,5	89,5	48,1	
Group VII	n	1	8	45	54	0	1	30	31	85
	%	1,9	14,8	83,3	63,5	0,0	3,2	96,8	36,5	

**TOTAL**	**n**	**8**	**29**	**224**	**261**	**7**	**16**	**212**	**235**	**496**
	**%**	**3,1**	**11,1**	**85,8**	**52,6**	**3,0**	**7,0**	**90,0**	**47,4**	

Table S3. *Participants' answers on ITEM#3, by medical specialty group and sex.*

MEDICAL SPECIALTY	***SEX***	***Female***			***Total female***	***Male***			***Total male***	***TOTAL***
	REPLY	***NO***	***I DO NOT KNOW***	***YES***		***NO***	***I DO NOT KNOW***	***YES***		
Group I	n	3	3	1	7	1	1	2	4	11
	%	42,9	42,9	14,2	63,6	25,0	25,0	50,0	36,4	
Group II	n	9	8	8	25	0	6	0	6	31
	%	36,0	32,0	32,0	80,6	0,0	100,0	0,0	19,4	
Group III	n	2	2	13	17	9	6	15	30	47
	%	11,8	11,8	76,4	36,2	30,0	20,0	50,0	63,8	
Group IV	n	6	5	4	15	7	7	9	23	38
	%	40,0	33,3	26,7	39,5	30,4	30,4	39,2	60,5	
Group V	n	3	3	3	9	7	2	8	17	26
	%	33,3	33,3	33,4	34,6	41,2	11,8	47,1	65,4	
Group VI	n	36	43	55	134	34	35	55	124	258
	%	26,9	32,1	41,0	51,9	27,4	28,2	44,4	48,1	
Group VII	n	16	12	26	54	11	11	9	31	85
	%	29,6	22,2	48,2	63,5	35,5	35,5	29,0	36,5	
TOTAL	n	75	76	110	262	69	68	98	235	496
	%	28,7	29,2	42,1	52,6	29,3	29,0	41,7	47,4	

Table S4. *Participants' answers on ITEM#4, by medical specialty group and sex.*

MEDICAL SPECIALTY	***SEX***	***Female***			***Total female***	***Male***			***Total male***	***TOTAL***
	REPLY	***NO***	***I DO NOT KNOW***	***YES***		***NO***	***I DO NOT KNOW***	***YES***		
Group I	*n*	*1*	*1*	*5*	*7*	*0*	*0*	*4*	*4*	*11*
	%	*14,3*	*14,3*	*71,4*	*63,6*	*0,0*	*0,0*	*100,0*	*36,4*	
Group II	*n*	*1*	*1*	*23*	*25*	*0*	*0*	*6*	*6*	*31*
	%	*4,0*	*4,0*	*92,4*	*80,6*	*0,0*	*0,0*	*100,0*	*19,4*	
Group III	*n*	*1*	*1*	*15*	*17*	*8*	*1*	*21*	*30*	*47*
	%	*5,9*	*5,9*	*88,2*	*36,2*	*26,7*	*3,3*	*70,0*	*63,8*	
Group IV	*n*	*3*	*3*	*9*	*15*	*8*	*1*	*14*	*23*	*38*
	%	*20,0*	*20,0*	*60,0*	*39,5*	*34,8*	*4,3*	*60,9*	*60,5*	
Group V	*n*	*2*	*2*	*5*	*9*	*0*	*1*	*16*	*17*	*26*
	%	*22,2*	*22,2*	*55,6*	*34,6*	*0,0*	*5,9*	*94,1*	*65,4*	
Group VI	*n*	*21*	*7*	*106*	*134*	*20*	*4*	*100*	*124*	*258*
	%	*15,7*	*5,2*	*79,1*	*51,9*	*16,2*	*3,2*	*80,6*	*48,1*	

**Group VII**	n	8	3	43	54	4	2	25	31	85
	%	14,8	5,6	79,6	63,5	12,9	6,5	80,6	36,5	
**TOTAL**	n	37	18	206	261	40	9	186	235	496
	%	14,1	6,9	79,0	52,6	17,2	3,8	79,0	47,4	

Table S5. *Participants' answers on ITEM#5, by medical specialty group and sex.*

MEDICAL SPECIALTY	SEX	Female			Total female	Male			Total male	TOTAL
	REPLY	NO	I DO NOT KNOW	YES		NO	I DO NOT KNOW	YES		
**Group I**	n	0	3	4	7	0	1	3	4	11
	%	0,0	42,9	57,1	63,6	0,0	25,0	75,0	36,4	
**Group II**	n	0	11	14	25	0	2	4	6	31
	%	0,0	44,0	56,0	80,6	0,0	33,3	66,7	19,4	
**Group III**	n	1	2	14	17	7	6	17	30	47
	%	5,9	11,7	82,4	36,2	23,3	20,0	56,7	63,8	
**Group IV**	n	1	8	6	15	7	8	8	23	38
	%	6,7	53,3	40,0	39,5	30,4	34,8	34,8	60,5	
**Group V**	n	1	5	3	9	3	8	6	17	26
	%	11,1	55,6	33,3	34,6	17,6	47,1	35,3	65,4	
**Group VI**	n	28	23	83	134	33	21	70	124	258
	%	20,9	17,2	61,9	51,9	26,6	16,9	56,5	48,1	
**Group VII**	n	5	19	30	54	5	13	13	31	85
	%	9,3	35,2	55,5	63,5	16,2	41,9	41,9	36.5	
**TOTAL**	n	36	71	154	261	55	59	121	235	496
	%	13,8	27,2	59,0	52,6	23,4	25,1	51,5	47,4	

Table S6. *Participants' answers on ITEM#6, by medical specialty group and sex.*

MEDICAL SPECIALTY	SEX	Female			Total female	Male			Total male	TOTAL
	REPLY	NO	I DO NOT KNOW	YES		NO	I DO NOT KNOW	YES		
**Group I**	n	3	1	3	7	2	0	2	4	11
	%	42,9	14,2	42,9	63,6	50,0	0,0	50,0	36,4	
**Group II**	n	14	7	4	25	5	0	1	6	31
	%	56,0	28,0	16,0	80,6	83,3	0,0	16,7	19,4	
**Group III**	n	12	2	3	17	17	4	9	30	47
	%	70,6	11,8	17,6	36,2	56,7	13,3	30,0	63,8	
**Group IV**	n	11	2	2	15	17	5	1	23	38
	%	73,4	13,3	13,3	39,5	74,0	21,7	4,3	60,5	
**Group V**	n	8	0	1	9	11	1	5	17	26

	%	88,9	0,0	11,1	34,6	64,7	5,9	29,4	65,4	
**Group VI**	n	93	10	31	134	73	14	37	124	258
	%	69,4	7,4	23,2	51,9	58,9	11,3	29,8	48,1	
**Group VII**	n	37	10	7	54	28	2	1	31	85
	%	68,5	18,5	13,0	63,5	90,3	6,5	3,2	36,5	
**TOTAL**	n	178	32	51	261	153	26	56	235	496
	%	68,2	12,2	19,6	52,6	65,1	11,1	23,8	47,4	

Table S7. *Participants' answers on ITEM#7, by medical specialty group and sex.*

		Female			Total Female	Male			Total Male	TOTAL
		NO	I don't know	YES		NO	I don't Know	YES		
**Group I**	n	0	1	6	7	0	1	3	4	11
	%	0,0	14,3	85,7	63,6	0,0	25,0	75,0	36,4	
**Group II**	n	2	1	22	25	0	1	5	6	31
	%	8,0	4,0	88,0	80,6	0,0	16,7	83,3	19,4	
**Group III**	n	0	0	17	17	2	0	28	30	47
	%	0,0	0,0	100,0	36,2	6,7	0,0	93,3	63,8	
**Group IV**	n	0	0	15	15	1	2	20	23	28
	%	0,0	0,0	100,0	39,5	4,3	8,7	87,0	60,5	
**Group V**	n	2	2	5	9	0	1	16	17	26
	%	22,2	22,2	55,6	34,6	0,0	5,9	94,1	65,4	
**Group VI**	n	9	6	119	134	9	7	108	124	258
	%	6,7	4,5	88,8	51,9	7,3	5,6	87,1	48,1	
**Group VII**	n	3	4	47	54	1	2	28	31	85
	%	5,6	7,4	87,0	63,5	3,2	6,5	90,3	36,5	
**TOTAL**	n	16	14	231	261	13	14	208	235	496
	%	6,1	5,4	88,5	52,6	5,5	6,0	88,5	47,4	

Table S8*: Participants' answers on ITEM#8, by medical specialty group and sex.*

		Female			Total female	Male			Total Male	TOTAL
		NO	Maybe	YES		NO	Maybe	YES		
**Group I**	n	0	4	3	7	0	2	2	4	11
	%	0,0	57,1	42,9	63,6	0,0	50,0	50,0	36,4	
**Group II**	n	1	5	19	25	1	1	4	6	31
	%	4,0	20,0	76,0	80,6	16,7	16,7	66,6	19,4	
**Group III**	n	0	1	16	17	4	7	19	30	47
	%	0,0	5,9	94,1	36,2	13,3	23,3	63,4	63,8	
**Group IV**	n	0	7	8	15	0	7	16	23	38

	%	*0,0*	*46,7*	*53,3*	*39,5*	*0,0*	*30,4*	*69,6*	*60,5*	
Group V	***n***	*0*	*4*	*5*	*9*	*3*	*2*	*12*	*17*	*26*
	%	*0,0*	*44,4*	*55,6*	*34,6*	*17,6*	*11,8*	*70,6*	*65,4*	
Group VI	***n***	*6*	*21*	*107*	*134*	*9*	*32*	*83*	*124*	*258*
	%	*4,5*	*15,7*	*79,8*	*51,9*	*7,2*	*25,8*	*67,0*	*48,1*	
Group VII	***n***	*4*	*4*	*46*	*54*	*2*	*9*	*20*	*31*	*85*
	%	*7,4*	*7,4*	*85,2*	*63,5*	*6,5*	*29,0*	*64,5*	*36,5*	
TOTAL	***n***	*11*	*46*	*204*	*261*	*19*	*60*	*156*	*235*	*496*
	%	*4,2*	*17,6*	*78,2*	*52,6*	*8,1*	*25,5*	*66,4*	*47,4*	

Table S9. *Participants' answers among 30 responders "No" to ITEM#8, by medical specialty group, motivation and sex.*

**MEDICAL SPECIALTY**		**Group I**		**Group II**		**Group III**		**Group IV**		**Group V**		**Group VI**		**Group VII**			**Total**	
**SEX**	**MOTIVATION**	n	%	n	%	n	%	n	%	n	%	n	%	n	%		n	%
**Female**	**I do not believe in gender medicine.**	0	0,0	0	0,0	0	0,0	0	0,0	0	0,0	3	0,0	0	0,0		3	27,3
	**I do not believe this is the best way to update.**	0	0,0	0	0,0	0	0,0	0	0,0	0	0,0	1	25,0	1	25,0		2	18,2
	**I am aware of the differences between sex and gender in medicine.**	0	0,0	0	0,0	0	0,0	0	0,0	0	0,0	0	25,0	1	25,0		1	9,1
	**Other**	0	0,0	1	100,0	0	0,0	0	0,0	0	0,0	2	50,0	2	50,0		5	45,4
**Total female**		0	0,0	1	50,0	0	0,0	0	0,0	0	0,0	4	66,7	4	66,7		11	36,7
**Male**	**I do not believe in gender medicine.**	0	0,0	0	0,0	2	50,0	0	0,0	1	33,4	1	50,0	1	50,0		6	31,6
	**I do not believe this is the best way to update.**	0	0,0	0	0,0	1	25,0	0	0,0	0	0,0	0	0,0	0	0,0		3	15,8
	**I am aware of the differences between sex and gender in medicine.**	0	0,0	0	0,0	0	0,0	0	0,0	1	33,3	1	50,0	1	50,0		5	26,3
	**Other**	0	0,0	1	100,0	1	25,0	0	0,0	1	33,3	0	0,0	0	0,0		5	26,3
**Total male**		0	0,0	1	50,0	4	100,0	0	0,0	3	100,0	2	33,3	2	33,3		19	63,3
**Total**		0		2		4		0		3		6		6			30	
